# Effective Differences between 2D and 3D Planned Brachytherapy in Lung Cancer: An Institutional Retrospective Study

**DOI:** 10.3390/medicina60030452

**Published:** 2024-03-08

**Authors:** Nensi Lalić, Marko Bojović, Olivera Ivanov, Jelena Ličina, Spasoje Popević, Mihailo Stjepanović, Daliborka Bursać, Ivica Lalić, Rade Milić, Sanja Tomić, Biljana Parapid, Aleksandar Anđelković

**Affiliations:** 1 Faculty of Medicine in Novi Sad, University of Novi Sad, Hajduk Veljkova 3, 21137 Novi Sad, Serbia; nensi.lalic@mf.uns.ac.rs (N.L.); olivera.ivanov@mf.uns.ac.rs (O.I.); jelena.licina@mf.uns.ac.rs (J.L.); daliborka.bursac@mf.uns.ac.rs (D.B.); sanja.tomic@mf.uns.ac.rs (S.T.);; 2 Clinic for Pulmonary Oncology, Institute for Pulmonary Diseases of Vojvodina, 21204 Sremska Kamenica, Serbia; 3 Clinic of Radiation Oncology, Oncology Institute of Vojvodina, 21204 Sremska Kamenica, Serbia; 4 Faculty of Medicine, University of Belgrade, 11000 Belgrade, Serbia; spasoje.popevic@med.bg.ac.rs (S.P.); biljana.parapid@med.bg.ac.rs (B.P.); 5 Clinic for Pulmonology, University Clinical Centre of Serbia, 11000 Belgrade, Serbia; 6 Faculty of Pharmacy, University Business Academy in Novi Sad, Trg Mladenaca 5, 21101 Novi Sad, Serbia; ivica.lalic@faculty-pharmacy.com; 7 Medical Faculty of the Military Medical Academy, University of Defence in Belgrade, 11000 Belgrade, Serbia; rade.milic1975@gmail.com; 8 Pulmonology Clinic, Military Medical Academy, 11000 Belgrade, Serbia; 9 Clinic for Cardiology, University Clinical Centre of Serbia, 11000 Belgrade, Serbia

**Keywords:** airway obstruction, brachytherapy, dyspnea, non-small-cell lung cancer, pulmonary atelectasis

## Abstract

*Background and Objectives*: Advanced lung cancer is usually manifested by endoluminal tumor propagation, resulting in central airway obstruction. The objective of this study is to compare the high dose rate brachytherapy treatment outcomes in non-small-cell lung cancer (NSCLC) depending on the treatment planning pattern—two-dimension (2D) or three-dimension (3D) treatment planning. *Materials and Methods*: The study was retrospective and two groups of patients were compared in it (a group of 101 patients who underwent 2D planned high-dose-rate endobronchial brachytherapy (HDR-EBBT) in 2017/18 and a group of 83 patients who underwent 3D planned HDR-EBBT between January 2021 and June 2023). *Results*: In the group of 3D planned brachytherapy patients, there was a significant improvement in terms of loss of symptoms of bronchial obstruction (*p* = 0.038), but no improvement in terms of ECOG PS (European Cooperative Oncology Group Performance Status) of the patient (*p* = 0.847) and loss of lung atelectasis (if there was any at the beginning of the disease) (*p* = 0.781). Two-year overall survival and time-to-progression periods were similar for both groups of patients (*p* = 0.110 and 0.154). Fewer treatment complications were observed, and 91.4% were in 3D planned brachytherapy (BT) patients. *Conclusions*: Three-dimensionally planned HDR-EBBT is a suggestive, effective palliative method for the disobstruction of large airways caused by endobronchial lung tumor growth. Independent or more often combined with other types of specific oncological treatment, it certainly leads to the loss of symptoms caused by bronchial obstruction and the improvement of the quality of life of patients with advanced NSCLC. Complications of the procedure with 3D planning are less compared to 2D planned HDR-EBBT.

## 1. Introduction

Lung cancer still has the highest morbidity and mortality incidence of all malignant diseases throughout the world, even though standard treatment modalities (surgery, chemotherapy, and irradiation) have been supplemented by novel treatment modalities including biological and immune therapy [1]. The five-year survival of NSCLC amounts to 26% [2]. 

In recent years, interventional bronchoscopy procedures have been further improved. This particularly applies to treatment procedures for lung cancer due to central airway obstructions (CAO). The CAO is defined as any disease localized in the trachea, main or intermediary bronchi, which may be detected by radiology or bronchoscopy examinations [3]. It is estimated that about 20–30% of lung cancer patients have the CAO, as well as about 40% of lethal outcomes are associated with the locoregional progression of the disease [4,5]. Interventional bronchoscopy is applied as a radical treatment option in early stages or as a palliative treatment of advanced lung cancer stages; it is considered the key treatment modality. Palliative bronchoscopic treatment procedures providing reopening of the airway may be classified as immediate-effect (laser, electrocautery, and argon plasma cauterization (APC)) [6] or delayed-effect ones as brachytherapy and photodynamic therapy (PDT). High-dose-rate endobronchial brachytherapy (HDR-EBBT) is the most frequently applied method to relieve respiratory symptoms in advanced lung cancer patients with an endobronchial tumor substantially obstructing the airway lumen. When atelectasis of the lungs and serious symptoms (hemoptyses, dyspnea, cough, and infectious syndrome symptoms) are involved, palliative radiotherapy is strongly indicated, primarily the HDR EBBT [7]. It may be applied simultaneously with other treatment modalities, including external beam radiotherapy (EBRT), chemotherapy, and biological or immune therapy [8]. Brachytherapy is administered as the low dose rate endobronchial brachytherapy (LDR-EBBT), leaving the introduced catheter in the airway for three days, or as the HDR-EBBT, which does not require the catheter to be kept longer in the airway [9]. In the bronchoscopy unit, using a flexible bronchoscope, the polytetrafluoroethylene catheter is introduced via the nasal pathway (whenever possible). The bronchial catheter is placed in the close vicinity of the tumor, with its distal point at least 1 cm from the lower edge of the tumorous infiltration (2 cm in ideal conditions). When necessary, more than one catheter may be introduced to optimize the dose distribution (tumors in the carina-2), or in case of multiple tumors. The irradiation planning pattern may be either two-dimension (2D) or three-dimension (3D). If the 2D imaging method is used, the radiation dose distribution around the catheter is determined according to the X-ray image in two different projections. The doze around the applicator itself is thus determined, missing precise information about the radiation dose in the target lesion and the organs close to the target lesion [10]. When using computerized tomography (CT) in radiation planning, the information about the applicator’s localization, target lesion, and surrounding organs at risk (OAR) is available, which helps to make the 3D radiation planning pattern [11]. The crucial objective of CT radiation planning is to achieve the optimal irradiation treatment dose in the target lesion while simultaneously reducing the radiation dose in the OAR and critical structures [12]. In curative brachytherapy, the radiation field is defined as a three-step process: the macroscopic tumor lesion is defined by diagnostic methods and is designated as the gross tumor volume (GTV), the clinical radiation field is designated as the clinical target volume (CTV), and finally, the planning target volume (PTV) is estimated 1 cm over and 1 cm under the CTV due to the accuracy of localization and the possible movement of the patient during transport. In this way, the tumor, the bronchial catheter position, and the OAR are visualized [12,13,14]. The most frequently applied dose regimens are 2 × 7 Gy; 2 × 7.2 Gy and 1 × 15 Gy. Using a bronchial catheter, the radiation source—Iridium-192 iso-tope—is delivered in the vicinity of the tumor [15]. In our study, we examined two groups of patients with either 2D or 3D HDR-EBBT planning, analyzing the differences between them in their clinical response (tumor reduction and atelectasis resolution), patients’ disease symptoms and performance status improvement, treatment-induced complications, the disease progression time, and the overall survival.

## 2. Materials and Methods

This study was retrospective and it was conducted at the Institute for Pulmonary Diseases of Vojvodina (IPBV). We analyzed 210 consecutive patients in two time periods (2017/18 and between January 2021 and June 2023), divided according to the method of planning for brachytherapy. Out of the total 118 patients who underwent 2D planning, 101 patients met the eligibility criteria for the retrospective analysis. Similarly, from the 91 patients who underwent 3D planning, 83 patients fulfilled the criteria to be included in the study. All patients in the study had a visible tumor in the trachea, main bronchi, or in the upper or lower bronchi of both sides of the lungs. Almost all the patients in both examined groups had primary lung cancer—NSCLC; 4 patients in the first group and 9 in the second group had metastatic bronchial tumors. Almost all patients of both examined groups had other types of specific oncological therapy with applied BT. 

Inclusion criteria were as follows:

1. Patients older than 18 years and younger than 85 years.
2. Patients with cytologically or histologically confirmed lung cancer of endobronchial localization in the trachea and main bronchi.
3. Patients treated with brachytherapy alone or brachytherapy combined with external beam radiotherapy, cytotherapy, or a combination of both, and immunotherapy.
4. Patients in whom the disease relapsed in the form of an endobronchial tumor after surgery.

Exclusion criteria were as follows:

1. Patients with severe chronic obstructive pulmonary disease, decompensated cardiomyopathy or malignant arrhythmia, and coagulopathy.
2. Patients with ECOG PS 3 or 4.
3. Patients with more severe psychiatric diseases in terms of uncooperativeness for performing bronchoscopy.
4. Patients with brachytherapy probe displacement.

For all patients, the recommendation for the implementation of BT was made by the Oncology Board of IPBV.

### 2.1. Treatment Details

After the application of the brachytherapy probe and the applied radiation therapy in two sessions of 7 Gy, each seven days apart, all patients were examined after 6 weeks for the loss or reduction in disease symptoms as well as the radiological response to the applied brachytherapy. For brachytherapy contouring, planning, and dose prescription, we followed the recommendations of the GEC-ESTRO Brachytherapy Guidelines [16]. Brachytherapy was implemented using the after-loading technique with an Ir-192 source on the GammaMed Plus machine (Varian Medical Systems, Palo Alto, CA, USA) at the Clinic for Radiation Oncology, Oncology Institute of Vojvodina. The time to progression (TTP) was observed in two years from the moment of application of the first probe, and the overall survival (OS) was monitored from the moment of diagnosis of the disease until the second follow-up period or the date of death according to the lung cancer registry of IPBV.

### 2.2. Statistical Analysis

Descriptive statistics were presented using frequency, percentage, minimum, maximum, mean, and standard deviation. For comparing categorical variables, the Chi-square test and Fisher’s exact test were utilized. TTP and OS were illustrated using Kaplan–Meier curves and analyzed using hazard ratio Cox regression analysis. The data were graphically presented using Microsoft Office 2021 software, while statistical analysis was conducted using IBM SPSS v26 and JASP v0.18.3.0. In the study, a statistical significance level of 0.05 was employed.

## 3. Results

The study included a total of 184 NSCLC patients. Of them, 101 patients constituted Group 1 submitted to 2D HDR EBBT planning, who received BT in the period January 2017–January 2018; Group 2 included 83 patients with 3D HDR EBBT planning, applied in the period January 2021–June 2023. The primary goal of the study was to establish the differences between the 2D and 3D treatment planning groups in airway disobstruction and atelectasis elimination (if involved), performance status improvement, and post-treatment registered complications. Secondary objectives were to establish the difference in the TTP, and the 24-month OS between the two groups of patients. The patient’s demographic characteristics, frequency of histology types, and the disease TNM stage (the tumor, node, and metastasis staging system, eighth edition of the TNM classification for lung cancer) were reviewed in the study (Table 1). The mean patient age in Group 1 was 63.8 yrs (38–85) ± 8.0, and the Group 2 patients were at the average age of 67.36 yrs (47–85) ± 7.11. No statistically significant age differences were registered between the two groups. Group 1 included 87 males and 14 females, while 65 subjects in Group 2 were males and 18 were females. No statistically significant differences in the patient’s gender were registered between the two groups (*p* = 0.163). Regarding the histological type, most patients in both groups had squamous lung cancer type—71 (70.3%) and 56 (63.9%) patients, respectively. Adenocarcinoma was involved in 17 (16.8%) and 18 (21.7%) Group 1 and Group 2 patients, respectively. Non-classified NSCLC was registered in less than 5% of patients in both groups. Other lung cancer types were slightly more common in Group 2 (9 or 10.8%) than in Group 1 (4 or 4%). No statistically significant differences in lung cancer histological types were registered between the two groups (*p* = 0.052). Regarding the TNM stage of the disease, seven (6.9%) patients in the 2D HDR EBBT planning group had a stage II disease, but only one patient had this disease in the 3D HDR EBBT planning group. The TNM stage IIIA of the disease in 2D/3D groups was registered in 16 (15.8%) and 6 (7.2%) patients of the 2D and 3D group, respectively; the most common IIIB stage was registered in 49 (48.5%) and 61 (73.5%) patients of the 2D and 3D group, respectively; the TNM stage IV of the disease was involved in 29 (28.7%) Group 1 patients and 15 (18.1%) Group 2 patients. There were significant differences in the TNM stage of the disease registered between the two groups (*p* = 0.004).

The features of the HDR EBBT procedure itself in the two examined groups of patients are reviewed in Table 2. Regarding the endobronchial tumor localization and the EBBT catheter position, the main bronchus was the most common position of the EBBT catheter in both groups: the right main bronchus (RMB) in 26 (25.7%) and 36 (43.4%) patients of the 2D and 3D group, respectively; the left main bronchus (LMB) in 29 (28.7%) and 23 (27.7%) patients of the 2D and 3D group, respectively. Other EBB catheter sites were less common in both groups. No statistically significant differences in the tumor localization and the EBBT catheter position were registered between the two groups (*p* = 0.076). Regarding the number of inserted EBBT catheters, most patients of both groups had inserted catheters delivered twice by two 7 Gy irradiation doses of, EBBT 95 (94.1%) pts of the 2D group and 74 (89.2%) pts of the 3D group. One dose of 7 Gy was received by 5 (5%) 2D group/4 (4.8%) 3D group pts. Two six-month separated brachytherapy courses, i.e., four catheter applications and a total of 28 Gy irradiation doses, were delivered to 1(1%)-2D/5 and (6%)-3D group patients.

### 3.1. Palliation Rate and Clinical Response

The airway obstruction due to symptoms and their disappearance after the applied EBBT in both groups is reviewed in Table 3. The symptom response was reviewed at the first control, four weeks after the applied brachytherapy. During that period, other oncologic treatment modalities were initiated in patients of both groups, except one in the 2D group. A significant symptom response was detected in both groups, The symptoms examined were cough, temperature, dyspnea, and hemoptysis, and there were a few patients in both examined groups who were asymptomatic. For all symptoms, except hemoptysis, there was a statistically significant difference in favor of the patients of the 3D planned EBBT group (*p* = 0.038; 0.009; *p* < 0.001). The performance status was monitored by ECOG PS (the criteria formulated by the European Cooperative Oncology Group PS), and it was found that most patients of both groups had scored 2 ECOG PS. A significant performance status improvement was registered in both groups after the HDR EBBT, but no statistically significant differences between the groups were registered in ECOG PS improvement (*p* = 0.847).

**Figure 1:**
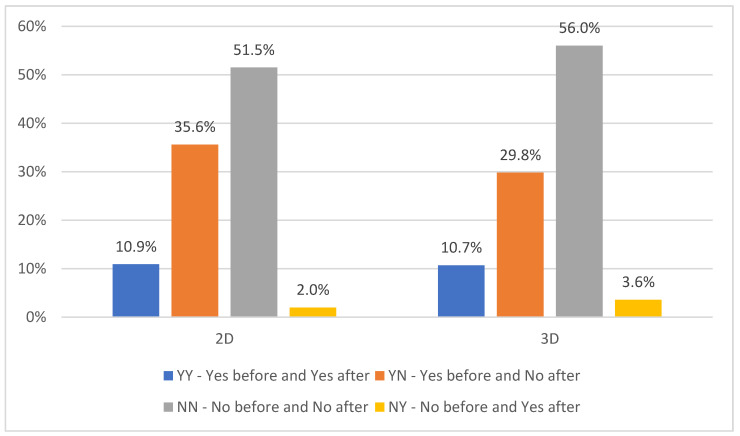
The presence and elimination of atelectasis related to 2D and 3D HDR-EBBT planning.

The local response to the applied brachytherapy (combined with other oncological treatment modalities) was examined by the criterion of the presence of atelectasis of the lobe or the entire lung and its elimination as a positive response to the treatment. The results are illustrated in Figure 1. Depending on the presence/absence of atelectasis of the lobe or the entire lung, the patients of both examined groups were classified as follows: YY—Yes before and Yes after EBB, YN—Yes before and No after EBB, NN—No before and No after EBB, and NY—No before and Yes after EBB. Comparing the two groups of patients, more patients with atelectasis were registered in Group 1, as well as those in whom atelectasis disappeared in response to the treatment. No statistically significant differences were found between 2D and 3D HDR EBBT planning groups (*p* = 0.781), as over 20% of the patients in both groups had atelectasis before but not after the treatment.

The complications due to treatment are reviewed in Table 4, including pneumothorax, hemoptyses, infectious syndrome requiring hospitalization, and parenteral antibiotic therapy. All the listed complications were less frequent in the 3D brachytherapy planning group, although no statistically significant difference between the examined groups was found.

### 3.2. Time to Progression and Overall Survival

**Figure 2:**
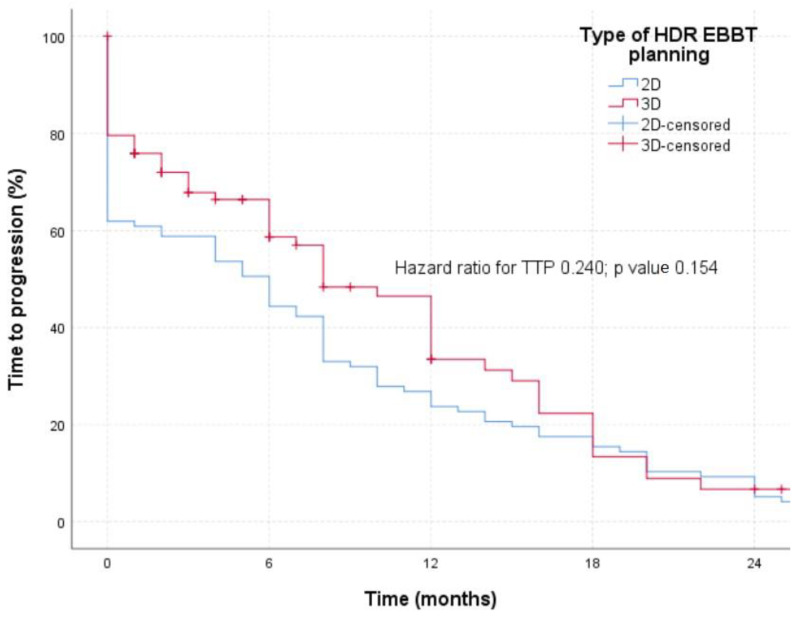
TTP in patients treated with 2D or 3D planned HDR-EBBT.

The TTP (Figure 2) was defined by the occurrence of the radiological progression seen in the chest CT finding, with a remark that brachytherapy was applied independently in patients neither in the group of 2D planned brachytherapy nor in the group of patients of 3D planned brachytherapy. No statistically significant difference in TTP was registered between the two examined groups (*p* = 0.154).

**Figure 3:**
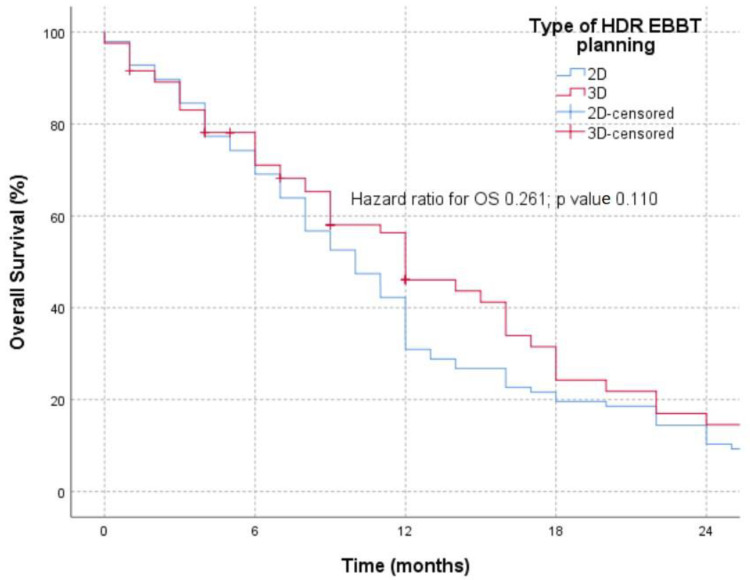
OS in patients treated with 2D and 3D HDR-EBBT planning.

The OS of the patients in the examined groups (Figure 3) was analyzed for 24 months because 3D planned brachytherapy was implemented in our hospital in 2021, and the group receiving 2D HDR-EBBT was treated in 2018. No statistically significant differences in the OS were registered between the two groups (*p* = 0.110), with a remark that stratification with other oncological treatment modalities was imprecise but uniform in both groups.

## 4. Discussion

Most NSCLC patients (about 70%) have an advanced stage of the disease at the time of diagnosis [17]. These patients are not candidates for surgery and require a multidiscipline approach to find out the best treatment modality to be applied. It is estimated that almost 30% of lung cancer patients have an obstruction due to a tumor in the bronchial tree, which is particularly important when localized in the trachea and main bronchi, defined as the CAO. About 40% of lethal outcomes in these patients are due to locoregional progression of the disease [3]. The CAO is due to the presence of the tumor in the trachea or the airway, external compression by the trachea/airway wall, or both of these tumor growth mechanisms. Depending on the obstructing mechanism, the adequate interventional therapeutic procedure is selected with a possible immediate effect (laser, electrocoagulation, APC, or a delayed therapeutic effect (cryotherapy, PDT, EBBT)) [18]. Brachytherapy is one of the oldest interventional bronchoscopy procedures, still applied as a palliative treatment procedure to reopen the bronchial tree. Although brachytherapy has been utilized in medicine since 1922, it started to be intensively applied in the treatment of lung cancer in the 1980s, when the technique and equipment for the high-dose (>12 Gy/h) after loading irradiation therapy with a precise irradiation field positioning were designed [19]. The radioactive source-iridium-192 is positioned via a formerly inserted catheter close to, or within the endobronchial tumor itself, delivering the necessary irradiation dose. HDR EBBT is a minimally invasive treatment procedure suitable for patients with a worse performance status, characterized by a short treatment duration, a low risk of catheter dislocation during the treatment, low costs, and no staff exposure to irradiation [20]. The disadvantages of this procedure include a delayed endoscopic treatment effect, occasional (although rare) acute complications (bleeding, pneumothorax, and infection), or late complications (airway stenosis or bronchial fistula). Due to a small irradiation volume and a rapidly declining irradiation dose with a growing distance from the irradiation source, reversely proportional to the airway lumen radius, this treatment modality is indicated for palliation of the airway obstruction due to symptoms (dyspnea, cough, hemoptyses, and fever due to post obstruction pneumonitis of the part of the lung which is not ventilated), and for reduction or elimination of atelectasis of the part of the lung [21]. Formerly applied 2D planning of the irradiation field based on the AP (anterior/posterior) and LL (lateral/lateral) X-ray does not provide a precise visualization of the tumor and the organs at risk or a precise endobronchial catheter positioning. Three-dimensional CT planning enables the irradiation dose to be applied on the PTV (GTV + 10 mm along the bronchial wall–CTV + 10 mm along the bronchial tree–PTV) margin or at 1 cm distance from the catheter center along the KK margin of 2 cm [22].

On the other hand, CT planning (identification of the applicator position and organs at risk (OAR), particularly blood vessels) reduces the possibility of complications, particularly hemoptyses. Direct contact between the applicator and the tracheobronchial walls closes large blood vessels, resulting in a great risk for hemoptyses. In 3D planning, the reference dose to entirely (100%) cover the PTV is 31% higher than in 2D EBBT, while the dose at OAR is at the same time minimized [23]. The primary goal of our study was to examine the effective differences between the NSCLC patients submitted to 2D or 3D HDR EBBT planning, including differences in symptom and performance status improvement, complications reduction, and local response to the treatment. There existed a difference in age between the two examined groups—the 2D HDR EBBT planning group was younger, which we could explain by the fact that patients from that period were diagnosed with advanced lung cancer when the process of screening diagnostics with low-dose chest CT had not yet been improved. In 2011, Aumont-le Guilcher et al. reported a median age of 62.2 years in their examined group of 226 patients receiving the 2D HDR EBBT planning [24], while Tamer Soror et al. reported a median age of 69 years of their patients receiving the 3D brachytherapy planning in which patients who received only brachytherapy were examined compared to patients who received brachytherapy with external beam radiotherapy [4]. In our study, no significant differences in gender were registered between the two examined groups, with the male gender predominating, which correlated well with other authors. Regarding the lung cancer histology, the squamous lung cancer type predominated in both examined groups; in addition, the 3D planning group included more patients with other lung cancer histology types (metastatic), suggesting that 3D planning, as a more recent method, has been increasingly applied for endobronchial metastases as well, further suggesting that the treatment of lung cancer has been improving in recent years, as well as the survival of oncologic patients, and the number of patients receiving the palliative treatments [25]. Squamous lung cancer was the dominant lung cancer type in both examined groups in our study, correlating with the results reported by other authors [26], explained by the fact this tumor type takes a central and endoluminal localization more frequently than other LC histology types [27]. In several studies, HDR EBBT has been proven an efficient palliative procedure, as well as an excellent procedure to achieve local control of the disease, providing longer survival in certain cases [4,28]. In our study, aimed at correlating and comparing the 2D and 3D EBBT planning, brachytherapy was used as a palliative treatment as the patients in both examined groups had advanced LC stage: stage IIIB predominated in the 3D EBBT planning (applied more recently) group, while more patients of the 2D EBBT planning (formerly applied) group had stage IV disease, suggesting that better control of the disease and fewer patients with metastatic disease are due to the newer 3D EBBT planning. Similar results (most patients with stage IIIB of the disease) were reported by Macías-Lozano et al. [26]. Regarding the most common endobronchial tumor localization, the main bronchi were most frequently involved in both groups of our study, correlating with other authors’ reports [29,30]. HDR EBBT dosing and fractionating are different for curative and palliative treatment. Most studies also reported different doses and fractions of palliative NSCLC brachytherapy as well [30,31,32]. In our study, the dose of 7 Gy in two 7-day separated fractions was applied in both the examined groups. If the tumor was localized on the main bifurcation—the trachea—two EBBT catheters were applied and inserted bilaterally in the main bronchi in two fractions. That was the situation in one and five patients of the 2D and 3D groups, respectively. 

The primary goal of our study was to define the differences between the 2D and 3D EBBT planning groups regarding the elimination of the bronchial obstruction symptoms. Former studies reported elimination of the bronchial obstruction after the applied brachytherapy in over one-third of the patients [33,34,35,36,37]. However, these studies miss the results regarding the obstruction symptom elimination due to 3D EBBT planning, which provides reliable information about the position of the applicator, target, and OAR on CT scans. In our study, due to these characteristics of the CT-planned brachytherapy, we have reported a significantly higher elimination of all bronchial obstruction symptoms than with the 2D EBBT planning. 

Brachytherapy represents a palliative procedure in LC where there is obstruction of the main airways by a tumor and is often used with other interventional therapeutic procedures, all to rapidly improve the Quality of Life (QoL) [38]. The effects of brachytherapy are significant because patients can have an improvement in QoL in a few hours or days after the applied procedure, and in most cases, brachytherapy has a palliative therapeutic goal in the advanced stage of the disease [13,39]. In our study, an improvement in the patient’s ECOG status was demonstrated for both types of applied brachytherapy.

In our study, we presented two-year overall survival for patients of both the investigated groups, as well as the period without disease progression. The comparison between the two groups of patients examined was quite solid, considering that approximately the same number of patients with brachytherapy received other treatment modalities (radiotherapy, chemotherapy, or both) in both groups of patients. Two-year survival in the group of 2D planned brachytherapy was 10.31%, and in the group of 3D planned BT, it was 15.2%. There was no statistically significant difference between the two groups of patients examined (*p*= 0.556; HR 0.100). Similar results were obtained for PFS between the two investigated groups of patients, where the PFS for 2D planned BT was 5.15%, and for 3D planned BT, it was 2.41% without a statistically significant difference (*p* = 0.154; HR 0.240). When looking at the results of other authors, in the work of Macías-Lozano, in patients in the initial stage of the disease, BT was applied curatively, and the two-year survival for these patients was 75% [26]. Guilcher et al. also showed a 2-year survival rate of 57% in patients with early-stage disease treated with BT [24]; the French group of researchers Marsiglia et al. applied BT at the initial stage of the disease and reported a 2-year survival result of 78% [40]; and Lorchel et al. reported 54% [41]. However, when it comes to the palliative application of BT in advanced-stage NSCLC, it is very difficult to compare the results of the survival rate because the indications for BT and the types of combined treatments were different. Thus, Harms et al. reported the survival rate of patients with advanced-stage NSCLC in whom there was a recurrence of endobronchial disease after only 5 months of EBRT [42]. Rochet et al. published the results of a retrospective study with 35 patients treated with HDR-EBBT and EBRT, with a median survival of 39 months and a 2-year survival of 61% [43]. Anacak et al. published a prospective study with 30 patients, with NSCLC in stage III disease, treated with HDR-EBBT and EBRT, where the median survival was 11 months, and the 5-year survival was 10% [44].

## 5. Conclusions

Three-dimensionally planned HDR-EBBT is a suggestive, effective palliative method for the disobstruction of large airways caused by endobronchial lung tumor growth. Independent or more often combined with other types of specific oncological treatment, it certainly leads to the loss of symptoms caused by bronchial obstruction and an improvement in the quality of life of patients with LC. Complications of the procedure with 3D planning are less compared to 2D planned HDR-EBBT.

## Figures and Tables

**Table 1:** Patients and tumor characteristics in 2D and 3D HDR EBBT planning groups.

		Type of EBBT Planning			
		2D	3D	∑	*p* Value
**Age**		63.8 (38–85) ± 8.03	67.36 (47–85) ± 7.11	65.42 (38–85) ± 7.81	0.001 ^a^
**Gender**	Male	87 (86.1%)	65 (78.3%)	152 (82.6%)	0.163 ^b^
	Female	14 (13.9%)	18 (21.7%)	32 (17.4%)	
**Histological type**	SCC	71 (70.3%)	56 (63.9%)	124 (67.4%)	0.052 ^c^
	ADC	17 (16.8%)	18 (21.7%)	35 (19%)	
	SCLC	5 (5%)	1 (1.2%)	6 (3.3%)	
	NSCLC/NOS	4 (4%)	2 (2.4%)	6 (3.3%)	
	other	4 (4%)	9 (10.8%)	13 (7.1%)	
**TNM**	II	7 (6.9%)	1 (1.2%)	8 (4.3%)	0.004 ^c^
	IIIA	16 (15.8%)	6 (7.2%)	22 (12%)	
	IIIB	49 (48.5%)	61 (73.5%)	110 (59.8%)	
	IV	29 (28.7%)	15 (18.1%)	44 (23.9%)	
**Other** **treatments**	CTH	67 (66.3%)	41 (49.4%)	108 (58.7%)	0.024 ^b^
	EBRT	49 (49%)	50 (60.2%)	99 (54.1%)	0.139 ^b^
	MTH	/	5 (6%)	5 (4.7%)	0.593 ^c^
	OP	3 (3%)	/	3 (1.6%)	0.253 ^c^
	MMT	45 (44.6%)	30 (36.1%)	75 (40.8%)	0.292 ^b^
**∑**		101 (100%)	83 (100%)	184 (100%)	

EBBT—endobronchial brachytherapy, TNM—tumor nodus metastasis staging, SCC—squamous cell carcinoma, ADC—adenocarcinoma, SCLC—small-cell lung carcinoma, NSCL—non-small cell lung carcinoma, NOS—non-classified, CTH—chemotherapy, EBRT—external beam radiation therapy, MTH—molecular therapy, OP—operative treatment, and MMT—multimodality treatment—combination of 2 or more therapies. ^a^ Independent Samples *t* Test; ^b^ Chi-Square Test; ^c^ Fisher’s Exact Test.

**Table 2:** Tumor location and number of fractions in 2D and 3D HDR EBBT planning groups.

		Type of EBBT Planning			
		2D	3D	∑	*p*-Value
**Location**	Trachea	16 (15.8%)	13 (15.7%)	29 (15.8%)	0.076 ^c^
	RMB	26 (25.7%)	36 (43.4%)	62 (33.7%)	
	IMB	4 (4%)	5 (6%)	9 (4.9%)	
	RUB	5 (5%)	/	5 (2.7%)	
	RMdB	3 (3%)	1 (1.2%)	4 (2.2%)	
	RLB	4 (4%)	1 (1.2%)	5 (2.7%)	
	LMB	29 (28.7%)	23 (27.7%)	52 (28.3%)	
	LUB	4 (4%)	/	4 (2.2%)	
	LLB	4 (4%)	2 (2.4%)	6 (3.3%)	
	BMB	6 (5.9%)	2 (2.4%)	8 (4.3%)	
**Number of fractions**	I	5 (5%)	4 (4.8%)	9 (4.9%)	0.179 ^b^
	II	95 (94.1%)	74 (89.2%)	169 (91.8%)	
	IV	1 (1%)	5 (6%)	6 (3.3%)	

EBBT—endobronchial brachytherapy, RMB—right main bronchus, IMB—intermediate bronchus, RUB—right upper bronchus, RMdB—right middle bronchus, RLB—right lower bronchus, LMB—left main bronchus, LUB—left upper bronchus, LLB—left lower bronchus, and BMB—both main bronchus. ^b^ Chi-Square Test; ^c^ Fisher’s Exact Test.

**Table 3:** Symptom response and ECOG changes before and after HDR EBBT.

		Type of EBBT Planning			
		2D	3D	∑	*p*-Value
**Asymptomatic**	YY	62 (61.4%)	37 (44.6%)	53.8%	0.038 ^b^
	YN	38 (37.6%)	41 (49.4%)	42.9%	
	NN	1 (1%)	4 (4.8%)	2.7%	
	NY	/	1 (1.2%)	0.5%	
**Cough**	YY	49 (48.5%)	18 (21.4%)	36.2%	<0.001 ^b^
	YN	43 (42.6%)	39 (46.4%)	44.3%	
	NN	5 (5%)	22 (26.2%)	14.6%	
	NY	4 (4%)	5 (6%)	4.9%	
**Temperature**	YY	7 (6.9%)	2 (2.4%)	4.9%	0.009 ^b^
	YN	16 (15.8%)	30 (35.7%)	24.9%	
	NN	72 (71.3%)	50 (59.5%)	65.9%	
	NY	6 (5.9%)	2 (2.4%)	4.3%	
**Dyspnea**	YY	49 (48.5%)	18 (21.4%)	36.2%	<0.001 ^b^
	YN	38 (37.6%)	28 (33.3%)	35.7%	
	NN	13 (12.9%)	30 (35.7%)	23.2%	
	NY	1 (1%)	8 (9.5%)	4.9%	
**Hemoptysis**	YY	5 (5%)	2 (2.4%)	3.8%	0.490 ^b^
	YN	15 (14.9%)	12 (14.3%)	14.6%	
	NN	75 (74.3%)	68 (81%)	77.3%	
	NY	6 (5.9%)	2 (2.4%)	4.3%	
**ECOG PS**	Same	45 (44.5%)	34 (40.5%)	42.7%	0.847 ^b^
	Better	53 (40.5%)	47 (56%)	54.1%	
	Worse	3 (3%)	3 (3.5%)	3.2%	
		101 (100%)	84 (100%)		

EBBT—endobronchial brachytherapy, 2D—two-dimensional (X-ray), 3D—three-dimensional (CT-based), and ECOG PS—Eastern Cooperative Oncology Group Performance Status. Presence of symptoms before and after brachytherapy: YY—Yes before and Yes after, YN—Yes before and No after, NN—No before and No after, and NY—No before and Yes after. ^b^ Chi-Square Test.

**Table 4:** Acute treatment complications.

	Type of EBBT Planning			
	2D	3D	∑	*p* Value
**Pneumothorax**	2 (2%)	2 (2.4%)	4 (2.2%)	1 ^c^
**Hemoptisis**	7 (6.9%)	5 (6%)	12 (6.5%)	1 ^c^
**Infective syndrome**	14 (13.9%)	10 (12%)	24 (13%)	0.716 ^b^
**Need for PTH AB**	8 (7.9%)	4 (4.8%)	12 (6.5%)	0.551 ^c^
**Pneumothorax cum drainage**	1 (1%)	-	-	-

EBBT—endobronchial brachytherapy, 2D—two-dimensional (X-ray), 3D—three-dimensional (CT-based), and PTH AB—parenteral antibiotic therapy. ^b^ Chi-Square Test; ^c^ Fisher’s Exact Test.

## Data Availability

The data presented in this study are available upon request from the corresponding author (accurately indicating status).
